# Ocular manifestations of vitamin A deficiency in gastrointestinal and hepatobiliary disease

**DOI:** 10.3389/fopht.2026.1779060

**Published:** 2026-04-29

**Authors:** Eric W. Lai, Timothy Lee, George Chen, Shih-Chung Lai, Paul Kang, Sidney A. Schechet

**Affiliations:** 1 Department of Ophthalmology and Visual Science, Yale School of Medicine, New Haven, CT, United States; 2 Department of Ophthalmology, Walter Reed National Military Medical Center, Bethesda, MD, United States; 3 Section of Digestive Diseases, Department of Internal Medicine, Yale School of Medicine, New Haven, CT, United States; 4 Department of Ophthalmology, Shuang-Ho Hospital, Taipei Medical University, New Taipei City, Taiwan; 5 Elman Retina Group, Baltimore, MD, United States

**Keywords:** corneal xerosis, dry eyes, gastrointestinal and hepatobiliary disease, nyctalopia, vitamin A deficiency, vitamin A deficiency retinopathy, xerophthalmia

## Abstract

**Purpose:**

To characterize clinical features, risk factors, and outcomes of vitamin A deficiency (VAD) among patients with gastrointestinal (GI) and/or hepatobiliary comorbidities.

**Methods:**

This retrospective study examined patients with GI or hepatobiliary disease who presented with ocular manifestations of VAD. Extracted data included patient demographics, underlying GI or hepatobiliary diagnoses, serum vitamin A levels, ophthalmic symptoms and examination findings, imaging features, and response to vitamin A repletion.

**Results:**

26 eyes from 13 patients were included. The most common systemic comorbidities were fatty liver disease (38.5%), prior gastric bypass surgery (23.1%), and cirrhosis (23.1%). Median serum vitamin A level at diagnosis was 19.1 mcg/dL (range, 2.5–36.1 mcg/dL, reference range 38–72 mcg/dL). Median presenting best visual corrected acuity (BCVA) was 20/50 (logmar 0.44, range, 20/20-light perception). Twenty-two eyes (84.6%) demonstrated anterior segment manifestations of VAD, including four eyes (15.4%) who presented with bilateral corneal ulceration and perforation. Posterior segment findings were present in 16 eyes (61.5%), including subretinal drusenoid deposits, RPE mottling, placoid macular changes, optic neuropathy, and a full-thickness macular hole. All patients reported bilateral vision loss, with 63.6% endorsing nyctalopia. All patients who underwent vitamin A repletion experienced subjective visual improvement and partial or complete resolution of ophthalmic findings, including regression of anterior and posterior segment pathology.

**Conclusions:**

Vitamin A deficiency occurs across a wide spectrum of GI and hepatobiliary diseases and is associated with diverse anterior and posterior segment ocular findings, ranging from subtle surface disease to severe, vision-threatening pathology. Visual symptoms and ocular findings improved following vitamin A repletion, highlighting the preventable and reversible nature of this condition when identified early. Given the risk of irreversible ocular sequelae with delayed diagnosis, serum vitamin A assessment with timely repletion should be considered in patients with at-risk gastrointestinal and hepatobiliary conditions. Early identification through multidisciplinary care may prevent avoidable, vision-threatening ocular complications.

## Introduction

Vitamin A is an essential fat-soluble micronutrient that plays a critical role in visual function and ocular surface health (1). Vitamin A is necessary for maintenance of conjunctival and corneal epithelial integrity, goblet cell differentiation, and immune defense of the ocular surface (2). In the retina, vitamin A derivatives are integral to the visual cycle, serving as precursors for 11-cis-retinal, the chromophore required for phototransduction in rod and cone photoreceptors (3–5). Deficiency disrupts rhodopsin regeneration, leading to impaired dark adaptation and nyctalopia, often the earliest clinical manifestation of vitamin A deficiency (VAD) (1, 3). As a result, vitamin A deficiency may produce a wide spectrum of anterior and posterior segment pathology, ranging from dry eye diseases with secondary complications to retinal dysfunction and irreversible vision loss.

Vitamin A homeostasis is regulated systemically and depends on adequate intestinal absorption, hepatic storage, and systemic storage, thus making patients with gastrointestinal (GI) and hepatobiliary disease particularly vulnerable to deficiency. Vitamin A is absorbed in the proximal small intestine in a bile-dependent process and subsequently stored in hepatic stellate cells (9–11). Disorders that impair fat absorption, bile secretion, or hepatic storage can significantly disrupt vitamin A metabolism (12–15). Gastrointestinal conditions associated with small intestinal mucosal injury, such as inflammatory bowel disease (IBD) and Celiac disease, as well as disorders characterized by impaired fat-digesting enzymes, including chronic pancreatitis and cystic fibrosis, can impair vitamin A absorption (16–18). In the post-bariatric surgery population, especially following Roux-en-Y gastric bypass, altered anatomy and malabsorption substantially increase the risk of fat-soluble vitamin deficiencies, including vitamin A (19–21). Chronic liver disease and cirrhosis reduce hepatic vitamin A storage capacity and impair retinol-binding protein synthesis, further exacerbating systemic deficiency (11, 22).

While VAD remains a leading cause of preventable blindness in developing regions due to inadequate dietary intake or malnutrition, the underlying etiologies differ in developed countries, where deficiency is more commonly associated with GI malabsorption, hepatobiliary dysfunction, and prior bariatric surgery (1, 6, 10). As a result, the clinical presentation and patient population may differ substantially between these settings. Despite this distinction, much of the existing literature has focused on nutritional deficiency in resource-limited regions, with comparatively fewer studies characterizing VAD in developed populations (16–19). This highlights the importance of recognizing alternative risk factors and clinical presentations in these settings.

Despite its well-established pathophysiology, VAD remains underrecognized in patients with GI and hepatobiliary disease, and screening practices are not well standardized nor avidly performed in comparison to other vitamin deficiencies. Reported prevalence of VAD varies widely depending on the underlying condition, ranging from 10-70% in post-bariatric surgery patients and up to 50% in patients with advanced liver disease (19, 23–26). However, routine screening for vitamin A levels is not uniformly recommended in clinical guidelines, and testing is often performed only after systemic or ocular symptoms develop (19). As a result, VAD may go unrecognized until advanced, vision-threatening manifestations develop, at which point ocular damage may be irreversible despite vitamin A repletion. Ophthalmic involvement is often overlooked, misdiagnosed, or misattributed to more common ocular surface or retinal disorders, further delaying diagnosis and treatment (27, 28).

Ocular manifestations of VAD are diverse and may involve both the anterior and posterior segments. Early anterior segment findings include conjunctival xerosis, decreased tear production, and punctate epithelial erosions (5, 6). Progressive deficiency may result in significant pathology such as corneal xerosis, nonhealing epithelial defects, stromal ulceration, keratomalacia, and, in severe cases, corneal perforation (6–8). Posterior segment involvement includes nyctalopia, visual field constriction, retinal pigment epithelium (RPE) dysfunction, and characteristic retinopathy with drusen-like deposits, placoid lesions, or outer retinal disruption on multimodal imaging (29–33). Importantly, many of these manifestations are potentially reversible with timely vitamin A repletion, emphasizing the importance of early recognition (1, 6).

Given the increasing prevalence of GI and hepatobiliary disease in the modern population and the absence of standardized screening protocols, VAD represents an underappreciated yet preventable cause of ocular morbidity. This multicenter retrospective study aims to characterize systemic risk factors, ophthalmic manifestations, and clinical outcomes of VAD in patients with GI and hepatobiliary comorbidities, with the goal of improving earlier recognition in this vulnerable population.

## Materials and methods

### Study design and ethics approval

This was a retrospective case series consisting of patients seen at two ophthalmology clinics and one tertiary hospital. Institutional Review Board (IRB) approval was waived at Yale University due to the retrospective nature of the study (#200040389). Patient information was gathered and secured in accordance with the Health Insurance Portability and Accountability Act, and the study followed the tenets set forth by the Declaration of Helsinski.

The electronic medical records of patients with a clinical diagnosis of VAD seen at Yale New Haven Hospital, Yale Eye Center, and Elman Retina Group, between January 2015 and December 2025, were reviewed. Candidate subjects were identified through querying local medical records database at these clinical sites for patients with a diagnosis of vitamin A deficiency and any GI or hepatobiliary diseases. Inclusion criteria included presence of a documented GI or hepatobiliary comorbidity, serum vitamin A levels outside of normal reference range, and ophthalmic examination findings or symptoms consistent with VAD. Patients without confirmatory laboratory testing or with alternative ocular diagnoses sufficient to explain their findings were excluded.

### Laboratory testing and analysis

Serum retinol levels were obtained through standard clinical laboratory blood testing as part of routine patient care. Testing was conducted by hospital-based and/or approved reference clinical laboratories, including Yale New Haven Hospital and Quest Diagnostics, using serum-based assays for vitamin A (retinol) quantifications that are cleared or approved by the U.S. Food and Drug Administration (FDA) and validated in accordance with Clinical Laboratory Improvement Amendments (CLIA) regulations for clinical diagnostic use.

Due to the retrospective nature of this study utilizing clinically reported laboratory values, the specific analytic platforms (chromatographic or other validated methodology), lower and upper limits of detection, calibration standards, and assay comparison with high-performance liquid chromatography (HPLC) were maintained by the performing laboratory under CLIA-certified quality assurance protocols and not available for further analysis. Detailed assay performance characteristics were not directly accessible to the study investigators and therefore were not independently analyzed. The reference range for serum retinol provided by the performing laboratory was 38–72 mcg/dL. All laboratory values were confirmed through electronic medical record review.

Serum retinol-binding protein 4 (RBP4) and beta carotene levels were not assessed in this study. RBP4 and beta carotene testing were not ordered by gastroenterologists and/or primary care providers as part of clinical care for these patients and serum samples were not available for additional *post hoc* analysis.

### Clinical diagnoses

Clinical diagnoses of GI and hepatobiliary conditions were established through detailed medical record review. Diagnoses were based on documentation by treating gastroenterologists or PCP’s and supported by relevant clinical data, including laboratory findings (e.g. elevated liver enzymes, abnormal autoimmune markers, etc.), imaging studies (ultrasound, CT, or MRI demonstrating pathology such as hepatic steatosis or structural abnormalities), and past surgical history (e.g. bariatric surgery). Patients were included as having a clinical diagnosis of GI and/or hepatobiliary condition if on chart review, at least three criteria were met. Clinical features of patients exhibiting ocular manifestations of VAD are detailed in this report.

### Medical records review

Electronic health records were reviewed for each patient. All patients had undergone complete ophthalmic examination. Collected data included patient demographics, underlying GI or hepatobiliary diagnoses, history of bariatric or intestinal surgery, and relevant systemic medical comorbidities. Laboratory data included serum vitamin A levels at the time of diagnosis and if available, serum levels post-repletion. Ophthalmic data included presenting symptoms, best-corrected visual acuity (BCVA), slit-lamp findings, dilated fundus examination findings, and available multimodal imaging, including optical coherence tomography (OCT), fundus photography, fundus autofluorescence (FAF), fluorescein angiography (FA), and electroretinograms (ERG). Visual acuity measurements were converted to logarithms of the minimum angle of resolution (logMAR) units for analysis. Longitudinal data were collected when available.

Primary outcomes were the spectrum and frequency of ophthalmic manifestations of VAD, categorized as anterior segment and posterior segment findings. Secondary outcomes included changes in serum vitamin A level, visual symptoms, BCVA, and improvement or resolution of ocular findings following vitamin A repletion.

Vitamin A repletion regimens were prescribed at the discretion of the treating physicians in collaboration with GI, hepatology, and/or primary care providers (PCP), and were administered via oral or parental routes depending on the severity of deficiency and underlying systemic disease. Follow-up duration and visit intervals varied among patients. Subjective improvement in visual symptoms and objective improvement in ocular findings on clinical examination and imaging was documented at subsequent visits.

## Results

A total of 13 patients and 26 eyes met inclusion criteria. Mean (range) patient age at initial presentation was 60.7 (31-83) years. Five (38.5%) patients were female. All patients had at least one underlying GI or hepatobiliary comorbidity (**Table 1**). The most common etiologies include fatty liver disease (38.5%), prior gastric bypass surgery (23.1%), and cirrhosis (23.1%). Other etiologies include Crohn’s disease (15.4%), autoimmune gastritis (15.4%), Celiac disease (7.7%), pancreatic insufficiency (7.7%), hepatocellular carcinoma (7.7%), and gastric cancer (7.7%). Two patients (15.4%) had risk factors of significant alcohol use.

**Table 1:** Patient demographic and clinical history.

Patient	Age (yrs)/Sex	GI/Hepatobiliary comorbidities	Other nutritional deficiencies requiring supplementation	Baseline serum retinol (mcg/dL)	Past ocular history
1	68/M	Cirrhosis, hepatosteatosis, alcohol use disorder	Folate	19.1	Nutritional optic neuropathy OU
2	31/F	Celiac disease, alcohol use disorder	Folate, B12, thiamine	4.0	None
3	62/M	Crohn’s disease	Vitamin D, B12	6.4	Exposure keratopathy, pseudophakia OU
4	68/F	Gastric bypass surgery (2004), hepatitis A and B	Folate, B12, thiamine	20.2	Exposure keratopathy, cataracts OU
5	44/M	Exocrine pancreatic deficiency, autoimmune gastritis	Folate, vitamin C, vitamin D, zinc	17.4	Exposure keratopathy, cataracts, pannus OU
6	45/F	Bariatric surgery	Folate, B12, zinc	2.5	N/A
7	66/M	Non-alcoholic steatohepatitis	Folate, vitamin D	36	Pseudophakia, LASIK, pathologic myopia OU
8	44/F	Bariatric surgery (2015), autoimmune hepatitis, non-alcoholic steatohepatitis	Vitamin B6, folate, vitamin D	30	Myopia OU
9	68/M	Hepatocellular carcinoma, cirrhosis	Vitamin D, folate, iron	6.2	Nonexudative macular degeneration, pseudophakia, glaucoma OU
10	71/M	Crohn’s disease, colectomy	Vitamin B12	21.6	Nonexudative macular degeneration, myopia, pseudophakia OU
11	57/F	Non-alcoholic steatohepatitis	N/A	12	Non-proliferative diabetic retinopathy with macular edema OU
12	83/M	Cirrhosis	Folate, vitamin D	19.7	Nonexudative macular degeneration with geographic atrophy OU, BRVO OS
13	82/M	Gastric cancer s/p resection, non-alcoholic steatohepatitis	Vitamin D	36	Optic neuropathy secondary to amiodarone, pseudophakia, Salzmann degeneration OU

Key: OU, both eyes; M, male; F, female.

At initial presentation, ocular symptoms included bilateral decreased vision (84.6%), dry eyes (76.9%), nyctalopia (63.6%), reduced color vision (63.6%), and eye pain (18.2%) (**Table 2**). Median logarithm of the minimum angle of resolution (logMAR) visual acuity at presentation was 0.44 (Snellen equivalent, 20/50, logMAR range, 0.00-2.70, Snellen equivalent range, 20/20-light perception).

**Table 2:** Initial presenting symptoms, exam, and imaging findings.

Patient	Presenting symptoms	Ishihara color plates	Anterior segment findings	Posterior segment findings	OCT	FA	ERG
1	Decreased vision, nyctalopia, dry eyes OU	0/15 OU	Conjunctival xerosis OU	Optic disc pallor, peripheral drusenoid deposits, RPE hyperpigmentation OU	RPE and photoreceptor layer loss, drusenoid deposits, RPE mottling OU	N/A	N/A
2	Decreased vision, nyctalopia, dry eyes OU	4/15 OU	Conjunctival xerosis OU	Temporal optic disc pallor, peripheral drusenoid deposits OU	Drusenoid deposits, RPE mottling OU	N/A	N/A
3	Decreased vision, nyctalopia OU	1/15 OU	Normal	Peripheral drusenoid deposits, RPE changes OU	Drusenoid deposits, outer retinal and photoreceptor disruption, RPE mottling OU	Areas of patchy hypoperfusion	Abnormal rod response
4	Decreased vision, eye pain, dry eyes OU	1/15 OU	1mm corneal ulceration and perforation OD, 3mm OS	Unable	Unable	N/A	N/A
5	Decreased vision, dry eyes, eye pain OU	Unable	Large neurotrophic ulcers OU	Unable	Unable	N/A	N/A
6	Decreased vision, dry eyes, eye pain OU	Unable	Opacified cornea with corneal perforation OU	Unable	Unable	N/A	N/A
7	Nyctalopia OU	Unable	Bitot spots, LASIK flap OU	Peripheral drusenoid deposits OU	Drusenoid deposits, RPE mottling OU	N/A	Reduced cone and red response OU
8	Nyctalopia OU	N/A	Normal	Normal	N/A	N/A	N/A
9	Decreased vision nyctalopia, dry eyes OU	0/15	Conjunctival xerosis OU	Placoid maculopathy, peripheral drusenoid deposits OU, macular hole OD	Stage 2 full-thickness macular hole OD, outer retinal atrophy and loss, drusenoid deposit, RPE mottling OU	Early hypofluorescence, late stippled hyperfluorescence OU	Extinguished rod and cone responses OU
10	Decreased vision, dry eyes, nyctalopia OU	1/15	Bitot spots OU	Peripheral drusenoid deposits, parafoveal geographic atrophy	Drusenoid deposits, outer retinal atrophy, RPE mottling OU	N/A	N/A
11	Decreased vision, dry eyes OU	N/A	Conjunctival xerosis OU	Scattered microaneurysms, roth spots, diabetic edema OU	Trace macular edema OU	N/A	N/A
12	Decreased vision, dry eyes OU	4/15 OD, 6/15 OS	Bitot spots	Peripheral drusenoid deposits, geographic OU	Drusenoid deposits, outer retinal atrophy, RPE mottling OU, CME OS	Leakage OS secondary to CME	N/A
13	Decreased vision, dry eyes, nyctalopia OU	1/15 OU	Bitot spots, conjunctival xerosis OU	Optic nerve pallor, placoid maculopathy, peripheral drusenoid deposits, RPE mottling OU	Drusenoid deposits, ellipsoid zone and outer nuclear layer with RPE mottling OU, CME OD	Late staining with placoid appearance OU	Reduced cone responses OU

Key: OD, right eye; OS, left eye; OU, both eyes; OCT, ocular coherence tomography; FA, fluorescein angiography; ERG, electroretinogram; mm, millimeters.

Baseline serum retinol levels were low in all patients. Among the 13 patients, the median (range) retinol level at diagnosis was 19.1 (2.5-36.1) mcg/dL (reference range 38–72 mcg/dL). Ten patients (76.9%) had serum vitamin A levels initially obtained by an ophthalmologist, leading to the diagnosis of VAD before follow-up with a PCP or GI/hepatobiliary specialist. Four patients (30.7%) had severe serum vitamin A deficiency, quantified as <10 mcg/dL. Eleven patients (84.6%) had concurrent nutritional deficiencies requiring supplementation, most commonly vitamins D (81.8%), B9/folate (72.7%), B12 (36.4%), B1/thiamine (27.3%), and zinc (18.2%). No patients had received vitamin A supplementation prior to presentation.

**Figure 1:**
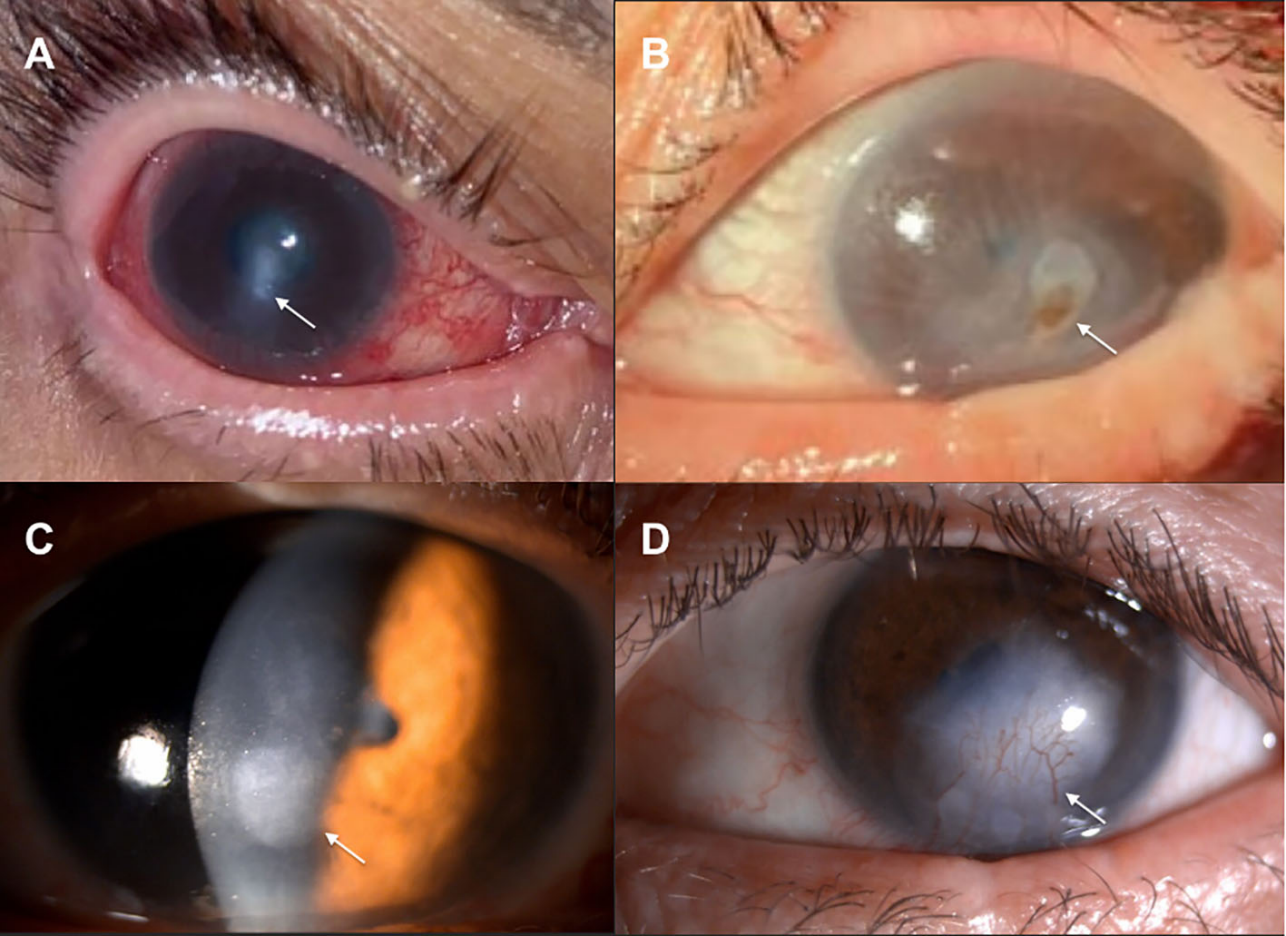
External photographs of patient #4 at initial presentation reveal acute bilateral corneal ulceration leading to perforation secondary to underlying vitamin A deficiency [**(A)**, arrow, right eye, **(B)**, arrow, left eye]. Slit-lamp photographs obtained one year after vitamin A repletion demonstrate healed perforation with corneal scarring in both eyes [**(C)**, arrow, right eye] and corneal neovascularization in the left eye [**(D)**, arrow].

On slit-lamp examination, 22 eyes (84.6%) demonstrated abnormal anterior segment findings. These included significant punctate epithelial erosions in 10 eyes (38.5%), Bitot spots in 8 eyes (30.8%), and non-healing epithelial defects in 6 eyes (23.1%). Two patients (15.4%) presented with severe bilateral corneal ulceration leading to perforation at time of diagnosis (**Figure 1**).

**Figure 2:**
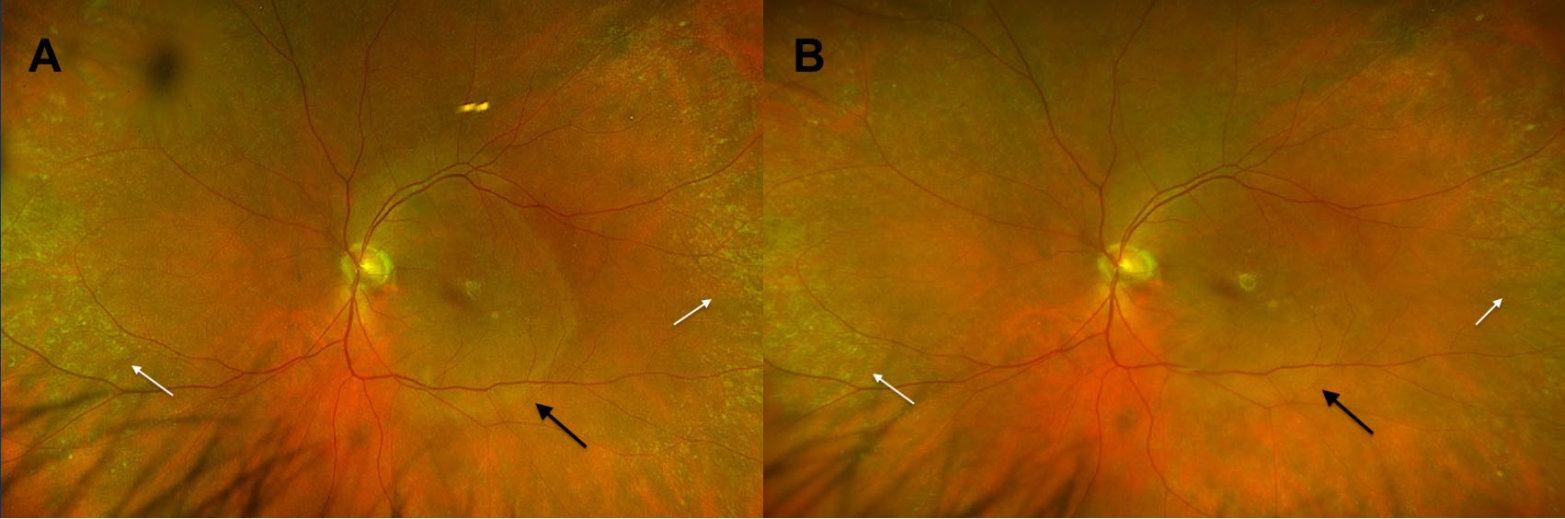
Wide-field fundus photographs of patient #9’s left eye at presentation demonstrate a well-circumscribed, placoid-like lesion in the posterior pole [**(A)**, black arrow] with surrounding peripheral yellow-white lesions resembling drusenoid deposits [**(A)**, white arrows] secondary to underlying vitamin A deficiency. Follow-up fundus photographs obtained two months after vitamin A repletion shows reduced prominence of the placoid-like lesion [**(B)**, black arrow] and drusenoid deposits [**(B)**, white arrows].

Posterior segment abnormalities were identified in 16 eyes (61.5%) on dilated fundus examination and ultra-widefield fundus photography. Findings included peripheral and macular drusenoid deposits (61.5%), RPE mottling (61.5%), optic nerve pallor (23.1%), placoid changes in the macula (15.4%), and a macular hole (3.8%) in one eye (**Figure 2**). In three patients, detailed posterior segment evaluation was not possible due to severe corneal ulceration or perforation, resulting in poor visualization on dilated examination and multimodal imaging. B-scan ultrasonography in these cases demonstrated a grossly flat retina without other acute posterior abnormalities.

Multimodal imaging was available in a subset of patients, including OCT in 9 patients, FAF in 6 patients, and FA in 4 patients. In 16 eyes (61.5%), OCT revealed subretinal hyperreflective deposits consistent with subretinal drusenoid deposits. Additional OCT findings included RPE mottling, ellipsoid zone loss, external limiting membrane defects, and RPE detachments in 12 eyes (52.5%). One eye (3.8%) demonstrated a stage 2 full-thickness macular hole at presentation. Six eyes (23.1%) with optic neuropathy demonstrated decreased nerve fiber layer (NFL) thickness, with average central thickness of 87 microns and temporal thickness of 41 microns. FAF imaging showed hypoautofluorescent foci colocalizing with subretinal hyperreflective deposits in 12 eyes (46.2%). FA findings included early hypofluorescence followed by late stippled hyperfluorescence consistent with placoid changes in 4 eyes. ERG data was completed for 4 patients prior to supplementation, with 8 eyes (30.8%) showing reduced or extinguished rod and cone responses on full-field ERG and/or reduced cone responses on multifocal ERG. Only one patient underwent repeat ERG post-vitamin A repletion, demonstrating normalization of rod and cone function (patient #9, **Table 2**), the rest declined testing or were lost to follow up after vitamin A repletion. Genetic testing was performed in two patients which was negative for inherited retinal dystrophy.

Mean (range) follow-up duration was 16.3 (1-36) months (**Table 3**). Seven patients received oral or parental repletion of vitamin A after diagnosis of VAD. Six patients were lost to follow-up before initiating supplementation. Patients received a medial initial loading dose of 100,000 international units (IU) daily, followed by dose at the discretion of treating physician. Among patients with follow-up after supplementation, median (range) post-repletion serum retinol levels increased to 47 (22-58) mcg/dL.

**Table 3:** Patient functional exam before and after repletion.

Patient	Initial supplementation regimen (IU)	Follow-up duration (months)	Final symptoms	Initial BCVA (OD, OS)	Final BCVA	Final serum retinol (mcg/dL)
1	100,000	3	Improved vision OU	20/100, CF@4ft	20/40, 20/150	58
2	N/A	N/A	N/A	20/50 OU	N/A	N/A
3	200,000	8	Improved vision and nyctalopia OU	20/25, 20/20	N/A	56
4	200,000	36	Healed corneal perforation, improved dryness and eye pain OU	20/400, Light perception	20/40, 20/150	31
5	100,000	1	Improving vision and eye pain OU	20/800, 20/400	20/100 OU	19
6	N/A	2	N/A	Light perception OU	LP, 20/400	N/A
7	N/A	36	N/A	20/30, 20/40	20/30 OU	N/A
8	N/A	1	N/A	20/20 OU	N/A	N/A
9	50,000	36	Improved vision, nyctalopia, and dry eyes OU	20/200, 10/100	20/30 OU	22.3
10	100,000	24	Improved nyctalopia	20/30, 20/25	20/30, 20/25	47
11	100,000	36	Improved vision OU	20/40, 20/30	20/30, 20/20	52
12	N/A	36	N/A	20/200, 20/80	20/200, 20/125	N/A
13	N/A	9	N/A	Counting fingers 3 feet, 20/100	Counting fingers 2 feet, 20/50	N/A

Key: IU, international units; OU, both eyes; OD, right eye; OS, left eye; LP, light perception; BCVA, best corrected visual acuity.

All seven patients who received vitamin A supplementation reported improvement or complete resolution of visual symptoms, including blurry vision, nyctalopia, color vision deficits, dry eye symptoms, and eye pain. All five patients with anterior segment pathology demonstrated improvement in ocular surface findings, including resolution of epithelial defects and/or healing of corneal perforations. All four patients with posterior segment involvement showed improvement in retinal findings post-repletion, including regression of drusenoid deposits, improvement in RPE mottling and placoid changes on fundus exam, and restoration of ellipsoid zone or ELM integrity on multimodal imaging. One eye demonstrated spontaneous closure of a full-thickness macular hole with normalization of rod and cone function on ERG following vitamin A repletion.

## Discussion

In this retrospective series, we describe a spectrum of ocular manifestations of vitamin A deficiency in patients with gastrointestinal and hepatobiliary disease, ranging from various ocular surface disease to posterior segment pathological findings. Our results highlight that VAD remains an underrecognized yet potentially reversible cause of vision loss, particularly among patients with malabsorptive, hepatic, or bariatric etiologies. To our knowledge, this represents the largest retrospective series focused specifically on ocular manifestations of VAD in a developed-country setting.

All patients in our cohort had at least one GI or hepatobiliary comorbidity known to impair vitamin A absorption, storage, or metabolism. Conditions such as bariatric surgery, chronic liver disease, inflammatory bowel disease (Crohn’s disease and ulcerative colitis), pancreatic insufficiency, and Celiac disease are well-established risk factors for VAD, particularly when accompanied by concurrent micronutrient deficiencies (34–37). Despite this recognized risk, screening for serum vitamin A levels remains non-standardized in these populations and is typically performed only in the setting of high clinical suspicion or after systemic or ocular manifestations have developed, in contrast to routine screening practices for other micronutrients.

For example, current guidelines recommend regular monitoring of vitamins B6, B12, and iron indices in patients with IBD (38). However, although VAD has been reported in more than 50% of patients with IBD and may exacerbate disease activity through immune dysregulation and increased intestinal permeability, vitamin A is not routinely assessed in this population (39). Consideration of vitamin A as part of standardized micronutrient evaluation in high-risk GI and hepatobiliary conditions may therefore facilitate earlier identification of deficiency, reduce preventable ocular morbidity, and potentially mitigate systemic disease progression. The high prevalence of concurrent nutritional deficiencies observed in our cohort highlights the systemic nature of malabsorption and supports a comprehensive approach to nutritional screening in these patients.

Anterior segment disease in our cohort was common and at times severe, with more than 80% of eyes demonstrating abnormal ocular surface findings. Punctate epithelial erosions, nonhealing epithelial defects, and Bitot spots were frequently observed, while two patients presented with bilateral corneal ulceration and perforation of unknown etiology until serum vitamin A level was obtained. Although corneal manifestations of VAD are well described, particularly in pediatric population, reports of corneal perforation in adults remain uncommon and are typically associated with delayed diagnosis (40–43). The absence of vitamin A supplementation prior to presentation in all patients suggests missed opportunities for earlier detection and intervention.

Additionally, posterior segment involvement was seen in more than 60% of patients in our cohort, including findings of subretinal drusenoid deposits, RPE mottling, placoid macular changes, optic nerve pallor, and in one case, a full-thickness macular hole. Prior literature describing retinal findings in VAD is limited largely to case reports and small series documenting nyctalopia, abnormal ERG responses, and nonspecific retinal changes (44–46). One case series of 9 patients by Levine et al. described multimodal imaging findings of subretinal hyperreflective deposits and external limiting (ELM) defects, with severe rod dysfunction on ERG in patients with VAD (47). Additionally, Bourke et al. described in another case series of 5 pediatric patients with VAD who presented with chronic optic neuropathy and subsequent irreversible vision loss despite vitamin A repletion (48). Our data is consistent and expands on these reports by demonstrating consistent structural correlates on fundus exam multimodal imaging.

The subretinal drusenoid deposits and placoid-like changes observed in this cohort may reflect disruption of retinoid cycling at the level of the RPE-photoreceptor interface. VAD impairs the visual cycle, leading to secondary photoreceptor dysfunction and outer retinal structural abnormalities, including ellipsoid zone and ELM disruption (47, 49–52). The frequent presence of rod and cone dysfunction on ERG further supports a functional correlate to these imaging findings.

Importantly, our data demonstrate that both anterior and posterior segment manifestations of VAD are at least partially reversible with appropriate supplementation. All patients who received vitamin A repletion experienced symptomatic improvement, accompanied by objective improvements in ocular surface integrity, retinal structure, and electrophysiologic function. Similar reversibility has been described in isolated reports, though documentation with contemporary multimodal imaging remains limited (47, 52–55).

Among the four patients with severe vitamin A deficiency, defined as serum retinol level <10mcg/dL, clinical presentations varied. Two patients had preserved visual acuity with relatively subtle anterior and posterior segment findings, whereas the remaining two presented with severe bilateral ocular involvement, including corneal perforations and placoid maculopathy with a full-thickness macular hole. These findings suggest that the severity of VAD did not consistently correlate with clinical severity in our cohort, although severe ocular manifestations were observed in half of patients with profound deficiency.

One particularly notable finding was the spontaneous closure of a full-thickness macular hole following vitamin A repletion. While macular hole formation has rarely been reported in association with VAD (33), this observation raises the possibility that metabolic dysfunction related to retinoid deficiency may contribute to retinal structural instability and that restoration of retinoid homeostasis may permit anatomical recovery.

This study is limited by its retrospective design, small sample size, and incomplete follow-up for some patients prior to supplementation. Additionally, multimodal imaging and electrophysiologic testing were not uniformly available for all patients. Lastly, serum vitamin A levels were obtained from routine clinical laboratory testing, and detailed assay-level performance characteristics were not available to the investigators.

Although the number of identified cases is modest, reported prevalence of VAD varies widely by underlying GI and hepatobiliary pathology, ranging from approximately 10%–70% among post–bariatric surgery patients and up to 50% in those with advanced liver disease (19, 23–26). In the United States, over 5 million adults are estimated to have significant chronic liver disease or cirrhosis – many at risk for impaired nutrient absorption – while several million have undergone bariatric surgery in the past decade. Applying the reported prevalence estimates to these populations suggests that hundreds of thousands of individuals could have VAD even before ocular symptoms arise (56, 57). Despite these substantial prevalence estimates, VAD remains underrecognized in routine clinical practice, as serum vitamin A levels are not commonly included in standard laboratory screening panels for at-risk patients. Systematic assessment in individuals with GI malabsorption or hepatobiliary dysfunction would likely reveal a meaningful proportion with biochemical deficiency prior to the onset of ocular manifestations. Thus, the relatively small number of cases included in this study likely reflects underdiagnosis and inconsistent screening rather than true rarity, underscoring a gap in current screening practices.

Our findings support a low threshold for targeted screening in high-risk populations. In particular, consideration of serum vitamin A assessment may be appropriate in patients with known GI and/or hepatobiliary disease who present with symptoms of unexplained progressive vision loss and/or nyctalopia, as well as exam findings of significant ocular surface disease and/or subretinal drusenoid deposits. In these settings, early identification of VAD may allow for timely intervention and prevent irreversible ocular sequelae. Larger prospective studies are needed to define the prevalence of deficiency in at-risk populations and to establish evidence-based screening strategies.

This series represents the largest cohort to date in literature describing ocular manifestations of VAD and underscores the need for broader, proactive testing to better define disease burden and facilitate earlier screening and intervention before irreversible ocular sequelae develop. Future studies are needed to evaluate the relationship between the severity of VAD and ocular signs, symptoms, and visual prognosis to identify thresholds associated with reversible versus permanent disease. Additionally, investigation into whether routine vitamin A supplementation, including as part of multivitamin regimens, can reduce deficiency risk in this high-risk population is warranted. Our small sample size and retrospective nature of this study limit our ability to propose formal screening guidelines. Thus, further studies are needed to define optimal dosing strategies, routes of administration, and safety considerations in these patients.

In summary, vitamin A deficiency associated with GI and hepatobiliary disease can lead to a broad spectrum of visually debilitating anterior and posterior segment pathology, many of which are reversible with timely recognition and treatment. However, delayed diagnosis may lead to irreversible ocular sequelae. In patients with at-risk gastrointestinal and hepatobiliary conditions, earlier consideration of serum vitamin A assessment alongside other nutritional and metabolic evaluations may facilitate detection of deficiency prior to the development of advanced ocular disease. When deficiency is identified, prompt vitamin A supplementation – tailored to the severity of deficiency – alongside treating the underlying GI or hepatobiliary comorbidity, may help mitigate ocular complications and prevent progression to permanent vision loss. Increased awareness and coordinated multidisciplinary care among primary care providers, gastroenterologists, hepatologists, and ophthalmologists may further improve recognition and management of vitamin A deficiency and reduce the risk of preventable vision loss in this vulnerable population.

## Conclusion

In this retrospective study, vitamin A deficiency was observed across a diverse spectrum of GI and hepatobiliary conditions, with patients most commonly presenting with symptoms of dry eyes, nyctalopia, and bilateral, progressive vision loss. Ocular findings spanned both anterior and posterior segment pathology, including dry eye disease, non-healing corneal ulcers or perforations, drusen-like deposits, outer retinal disruption, optic neuropathy, and placoid maculopathy. Among patients who underwent vitamin A repletion, improvement in ocular signs and symptoms was observed in all cases, underscoring the potential reversibility of disease with timely recognition and treatment. Collectively, these findings emphasize the need to maintain a high index of suspicion for vitamin A deficiency in patients with significant gastrointestinal or hepatobiliary disease. Proactive screening and timely vitamin A supplementation in this high-risk population may prevent irreversible ocular damage and improve visual outcomes.

## Data Availability

The raw data supporting the conclusions of this article will be made available by the authors, without undue reservation.
